# Development and validation of a knowledge-based model for robotic radiosurgery planning for brain lesions

**DOI:** 10.1016/j.phro.2026.101020

**Published:** 2026-06-11

**Authors:** Rita Buono, Roberta Castriconi, Alessia Tudda, Carmen Gigliotti, Lucia Perna, Paola Mangili, Antonella del Vecchio, Claudio Fiorino, Sara Broggi

**Affiliations:** a Medical Physics, IRCCS San Raffaele Scientific Institute, Milan, Italy; b Scuola di Specializzazione di Fisica Medica – Università degli Studi di Milano, Milan, Italy

**Keywords:** Robotic Radiosurgery, Knowledge-Based model, Dose gradients prediction

## Abstract

**Background and purpose:**

One of the main challenges in stereotactic planning is achieving a steep dose gradient to spare nearby structures. This study aimed to develop and validate a knowledge-based (KB) model for automated planning of single brain lesions using a robotic stereotactic system, achieving plan quality comparable or superior to manual plans.

**Materials and methods:**

Sixty retrospective plans were used to train the model. A relationship was established between the Planning Target Volume (PTV) radius and the effective radii of multiple isodose volumes. These regressions were used to generate patient-specific dose shell structures and automated optimization templates. Model performance was assessed through internal (15 cases) and external (13 cases) validation. Conformity Index (CI), Dose Gradient Index (DGI), healthy brain dose, and delivery parameters were compared between KB-generated and clinical plans.

**Results:**

The predictive accuracy of the model was ≥0.98 for all isodose levels. For both validation cohorts, KB plans achieved a significantly steeper dose fall-off. A median DGI of 87.3 vs 81.5 (*p* = 0.001) and of 88.5 vs 79.2 (*p* < 0.0001) was found, respectively. A significantly (*p* = 0.01) lower CI was found for external validation and a similar CI (*p* = 0.08) for the internal cohort. Automatic plans reduced irradiated healthy brain volume, particularly at 50% and 30% isodose levels. Significantly fewer beams were obtained: −58 (*p* = 0.004) and − 34 (*p* = 0.03), respectively for both cohorts.

**Conclusions:**

The proposed KB model enables automated stereotactic planning with high quality, efficiency, and standardization.

## Highlights

• A Knowledge-based model for automatic robotic radiosurgery planning was developed.
• Based on target volume, dose fall-off was predicted with R^2^ ≥ 0.98.
• Automatic plans had steeper dose gradients (Dose Gradient Index: 88.5 vs 79.2).
• Automatic plans achieved better conformity index (1.10 vs 1.24).
• Improved efficiency (−34 beams) and lower variability (−40%) with automated plans.

## Graphical abstract

**Figure:**
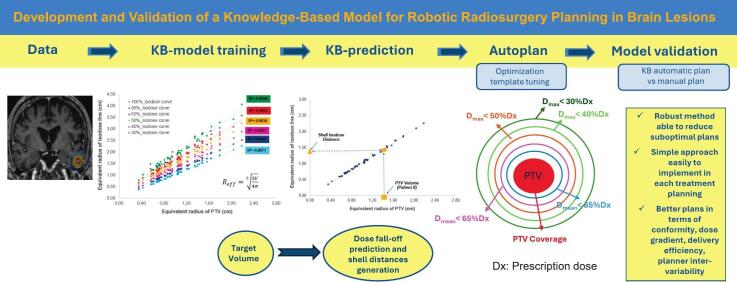

## 1 Introduction

Stereotactic Radiosurgery (SRS) and fractionated Stereotactic Radiotherapy (SRT) are advanced radiation therapy techniques that deliver high doses of radiation with submillimeter precision. A single high-dose fraction is delivered with SRS, while SRT uses multiple smaller fractions, making it suitable for larger targets or for lesions near critical structures. Both approaches aim to maximize tumor control while minimizing radiation exposure to healthy tissues and organs at risk (OARs) [1], [2], [3], [4], [5].

Radionecrosis (RN) is the primary complication associated with SRS/SRT, resulting from healthy brain tissue being irradiated [6]. Therefore, one of the primary challenges in SRS and SRT planning lies in achieving a steep dose gradient around the Planning Target Volume (PTV), ensuring a rapid dose fall-off in order to protect nearby critical structures [7], [8], [9], [10], [11].

The ability to rapidly deliver highly conformal dose distributions while minimizing irradiation of surrounding healthy tissues is critical, yet strongly dependent on planner experience [12], [13] and optimization strategy. Knowledge-Based (KB) planning has emerged as a promising solution to reduce planning time, improve consistency, and mitigate inter-planner variability by learning from previously high-quality treatment plans [14]. However, current KB planning approaches still face several limitations. Models are highly dependent on institution-specific datasets, are not easily transferable across centers or platforms, and often rely on complex predictors that are difficult to relate directly to target geometry or dose gradient behavior. This is particularly true in robotic stereotactic platforms, where there is a significant optimization freedom although this is difficult to standardize.

Multicenter studies [15], [16] have shown that geometric features, such as the effective radii of isodose volumes, strongly correlate with dose fall-off performance. Landoni et al. [15] highlighted the potential of multicenter predictive models to streamline the planning process and enhance inter-institute consistency of dose falloff.

Building on this concept, the aim of the current study was to develop and clinically implement a robust KB planning methodology for a robotic stereotactic platform, specifically focused on single brain lesions. The proposed approach leverages the relationship between the equivalent radius of the PTV and the surrounding body volumes receiving specific dose levels (isodose shells) to predict dose distribution parameters and drive automated plan optimization. By directly targeting the control of dose gradients, this planning approach seeks to overcome current KB planning limitations and to demonstrate equivalence/superiority compared with expert manual planning.

## 2 Materials and methods

### 2.1 Patient data

The current study was part of the Multi-Institutional Knowledge-based Approach to Planning Optimization for the Community project (MIKAPOCo), approved by the Institutional Review Board (IG23150, 248/2021). Eighty intracranial SRS/SRT treatment plans with single brain lesions from 75 patients, treated at our institute using the CyberKnife® System M6 (CK) (Accuray Inc., Sunnyvale, CA, USA) between 2018 and 2023, were randomly selected.

All plans involved single brain lesions far from critical structures, with no overlap or relevant proximity to OARs. Thus, OAR dose constraints did not limit optimization or affect the dose gradient outside of the PTV. For patients with multiple lesions, plans were included only if the optimization of one lesion did not influence the optimization of the other one.

Patients were treated with 1–5 fractions with prescribed doses ranging from 14 to 40 Gy. To generate an optimal KB model, a subgroup of 60 plans with Conformity Index (CI) ≤ 1.2 [16] were selected. A CI ≤ 1.2 is considered an ideal criterion in robotic radiosurgery planning [17], [18], [19] and was required by the Radiation Therapy Oncology Group (RTOG), specifically in RTOG 0915 and RTOG 0813 [20], [21].

The treatment plans were generated using fixed cones (22 plans; 5–12.5 mm) and IRIS™ variable aperture collimator (38 plans; 5–35 mm). Both sequential optimization and Volumetric Optimization (VOLO™) algorithms (Accuray Inc., Sunnyvale, CA, USA) were utilized, with dose calculation performed using a ray tracing algorithm.

Selected geometric, dose/volume metrics and planning information were recovered (Table S1), such as the body volumes enclosed in 100%, 85%, 65%, 50%, 40% and 30% isodose lines, with 100% corresponding to the prescription dose. The equivalent radius of volumes (R_eff_) defined by a set of isodose curves was then estimated as:

(1) $$\;R_{\mathrm{eff}}=\sqrt[{3}]{\frac{3V}{4\pi }}$$

where V identifies the body volume enclosed in selected isodose lines.

### 2.2 Knowledge based model development and template optimization

Based on the method suggested by Yu et al. [22], a KB model was developed by fitting the relationship between the PTV equivalent radius (R_*eff*_PTV) and the equivalent radius of the volume receiving a specified percentage of the prescription dose (R_*eff*_isodose). A linear relationship between R_*eff*_isodose and R_*eff*_PTV was previously demonstrated [17], according to:

(2) $$R_{\mathrm{eff}}\mathrm{isodose}=a∙R_{\mathrm{eff}}\mathrm{PTV}+b$$

where *a* and *b* represent the slope and offset of the fitted line, and $R_{\mathrm{eff}}$isodose and R_*eff*_PTV represent the radius of a sphere with geometric volume equal to a specific isodose and the PTV radius, respectively. The linear fit was built using the body volumes collected for 100%, 85%, 65%, 50%, 40% and 30% isodose lines, chosen to accurately describe the dose fall-off outside of the target volume. The goodness of fit was measured by the coefficient of determinant R^2^.

Once the model parameters *a* and *b* were assessed, they were used to predict the radii of isodose shells for new patients. An Excel-based tool was developed to implement this procedure. By entering the patient's PTV volume, the corresponding shell radii and distances for predefined dose levels could then be calculated.

An optimization template was then fine-tuned to replicate the planning strategies typically employed by expert planners using the VOLO optimization platform (Accuray Precision v 3.3). For each plan, 100% of the PTV received 100% of the prescribed dose, with a maximum dose ≤125%. To reinforce coverage, 98–99% of the PTV was required to receive a dose slightly above prescription (1–2 Gy more) (Table S2).

Only the isodose shells predicted by the KB model were defined as critical structures. Mean dose objectives were applied to the 85%–65% isodose shells, while maximum dose constraints were applied to the outer shells (50%–30%). Objectives were iteratively weighted to balance target coverage, dose conformity and dose fall-off, aiming to optimize both the CI and Dose Gradient Index (DGI) [16], [23]. The model was finally tuned to maximize CI and DGI, using variable prescription isodoses (typically ranging between 75% and 80%). A comparison between clinical and KB-based maximum doses was added to confirm the robustness of the model.

Conformity index and dose gradient index were computed from the collected data, using the following formulae:

(3) $$\mathrm{CI}=\frac{\mathrm{Prescription Volume}\;\left(\mathrm{cc}\right)}{\mathrm{Target Volume}\;\left(\mathrm{cc}\right)}$$

(4) $$\mathrm{DGI}=100-\left\{100∙\left(\left(R_{\mathrm{eff}},50\%R_{x}-R_{\mathrm{eff}},R_{x}\right)-0.3\;\mathrm{cm}\right)\right\}$$

### 2.3 Model validation

Two validation phases assessed the KB model. First, internal validation used 15 plans from the training set to test robustness and template adequacy. Second, external validation involved 13 new patients with single brain lesions to evaluate generalizability.

In both cases, complete automatic treatment plans were generated using the KB methodology, without any additional manual intervention from the planner and compared to the previously delivered clinical plans. The KB automatic plans were generated by using the same collimators (type and dimensions) used in the corresponding clinical plan. The PTV coverage, PTV maximum dose, CI, DGI, healthy brain dosimetry and selected technical CK machine parameters (including number of beams, number of nodes, monitor units (MU), and total estimated treatment time) were estimated and compared. Additional analyses in terms of new Conformity Index (nCI) are reported in the Supplementary Material A.

The paired *t*-test was used to compare data. All tests were two-sided, and a significance level of 0.05 was considered. Results with *p*-value <0.05 were regarded as statistically significant.

### 2.4 Clinical implementation

After validation, the KB model was clinically introduced. An additional shell, not predicted by the KB model, was placed 40–50 mm from the PTV and included in the optimization template, with a maximum dose constraint of 10% of the prescription to limit low-dose spread.

Optimization was first automatic, with optional manual adjustment. Only objective weights could be modified to improve PTV coverage or reduce healthy brain dose. Knowledge-based predicted shell distances and relative isodose levels remained fixed.

## 3 Results

**Fig. 1:**
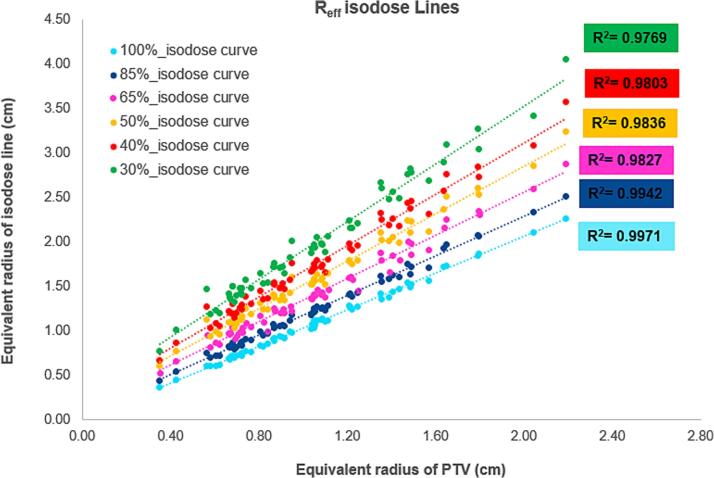
Linear regression between PTV equivalent radius and isodose shell radii at different dose levels.

The median PTV was 4.1 cm^3^ (range: 0.2–43.9 cm^3^) and the median distance between the PTV border and the nearest critical structure (i.e., optical structures, brain stem) was 38.4 mm (range: 5.2–98 mm). In Fig. 1, the linear relationships between R_eff_isodose and R_*eff*_PTV are shown. The corresponding intercept (b) and slope (a) coefficients are reported in Table S3.

The resulting R^2^ were ≥ 0.98, ranging from 0.977 to 0.997. During the tuning phase, a template consisting of 5 shells corresponding to R_eff_ of different isodoses values (85%, 65%, 50%, 40% and 30%) for the objectives of critical structures and one PTV coverage/inhomogeneity dose constraint was built and used for the validation phase. Details of the optimization template are reported in the Supplementary Material (Table S2).

For the internal cohort a similar PTV coverage (*p* = 0.15) was estimated between clinical and automatic KB plans: a median PTV coverage equal to 98.4% (94.9–100%) was found against 98.8% (96.5–100%). Knowledge-based plans exhibited a slightly lower prescription isodose level, with a median value of 78% (range: 67%–80%), compared to a median of 80% (range: 69%–80%) for clinical plans. Similarly, the maximum PTV dose showed comparable values between KB and clinical plans. A slightly lower CI was observed for KB automatic plans (1.05 ± 0.04) compared to clinical plans (1.09 ± 0.07), although the difference was not statistically significant (*p* = 0.08), indicating comparable conformality. A significantly higher DGI was found in KB-generated plans (*p* = 0.001), with a median value of 87.3 (range: 71.9–99.1) compared to 81.5 (range: 60.7–96.5) in clinical plans. In Table 1, the difference between the irradiated brain tissue volume (brain volume outside the Clinical Target Volume (Brain-CTV)) was reported at different isodose levels. The average results indicate a reduction in healthy brain tissue volume for KB plans.

**Table 1:** Volume average differences (cm^3^) for healthy brain (Brain–CTV) at different isodose levels between optimized KB and manual clinical plans.

	Mean difference (cm^3^)	p - value
Brain-CTV_100%	0.02 ± 0.5	0.88
Brain-CTV_80%	−0.20 ± 0.9	0.41
Brain-CTV_50%	−0.9 ± 1.5	0.04
Brain-CTV_30%	−1.4 ± 3.7	0.16

A mean reduction of 12 nodes (range: −90 – 50; p = 0.17), 58 beams (range: −209 - 5; p = 0.004) and 5 min (range: −30 - 6 min; *p* = 0.047) was found compared to clinical plans.

In the external cohort a similar (*p* = 0.12) PTV coverage was found between clinical and KB plans: a median PTV coverage of 99.9% (range: 95.8–100%) and 98.6% (range: 96.9–99.7%) was respectively found. A significant (*p* = 0.002) reduction of the prescription isodose level was found for KB plans (median value: 77% (range: 70–80%) vs 80% (range: 70–80%)). A significant (p = 0.002) increase of the PTV maximum dose was also found for KB plans (median value: 33.3 Gy (range: 28.6–44.9 Gy) vs 30.0 Gy (range: 27.5–44.9 Gy)). Most of KB-model plans provided significantly lower CI values (1.10 ± 0.06 vs 1.24 ± 0.17; *p* = 0.01) and significantly higher DGI values (DGI: 88.5 ± 15.5 vs 79.2 ± 17.9; *p* < 0.0001). In Table 2, the differences for Brain–CTV volume at different isodose levels are reported.

**Table 2:** Volume average differences (cm^3^) for healthy brain (Brain–CTV) at different isodose levels between optimized KB and manual clinical plans.

	Mean difference (cm^3^)	p - value
Brain-CTV_100%	−0.30 ± 0.7	0.17
Brain-CTV_80%	−1.1 ± 1.80	0.06
Brain-CTV_50%	−2.5 ± 4.2	0.05
Brain-CTV_30%	−4.1 ± 9.0	0.13

A significant average reduction of 13 nodes (range: −58–9; p = 0.03) and 34 beams (range: −162–19; *p* = 0.03) was found and an average reduction of 4 min of the treatment time (range: −21–11 min; *p* = 0.2) was also found.

21 patients with a single brain lesion with a median PTV volume of 2.9 cm^3^ (range: 0.4–34.7 cm^3^) were treated according to the proposed KB approach, with the prescribed dose range between 12 Gy and 40 Gy delivered in 1 to 5 fractions. Mean PTV coverage was 98.6% (range: 95.6–100%). Similar to the results of the external validation, the average isodose prescription level was 77.7% (range: 71%–82%) lower compared to previous clinical plans where for most of the plans the isodose level prescription was 80%. All of the KB-model plans showed CI < 1.2, (mean:1.07, range: 1.06–1.2).

When lesions were stratified according to the PTV volume groups defined by Landoni et al. [15], all plans showed a DGI value that was larger than the average reported in that particular study. A summary of the results is shown in Table 3.

**Table 3:** Average DGI and % of plans with DGI larger than DGI_80 for different lesion volumes. Average DGI and DGI_80% are defined in [15].

Group	DGI average [15]	DGI average	% plans DGI_80%
I (0–1 cm^3^)	94	98.4 (94.5–100.6)	50% (4 plans)
II (1–3 cm^3^)	84	94.1 (90.4–98.02)	100% (7 plans)
III (3–5 cm^3^)	78	86.2 (83.8–90.8)	33.3% (3 plans)
IV (5–10 cm^3^)	72	83	100% (1 plans)
V (10–15 cm^3^)	59	Na	Na
VI (>15 cm^3^)	50	64.8 (56.6–71.3)	50% (3 plans)

**Fig. 2:**
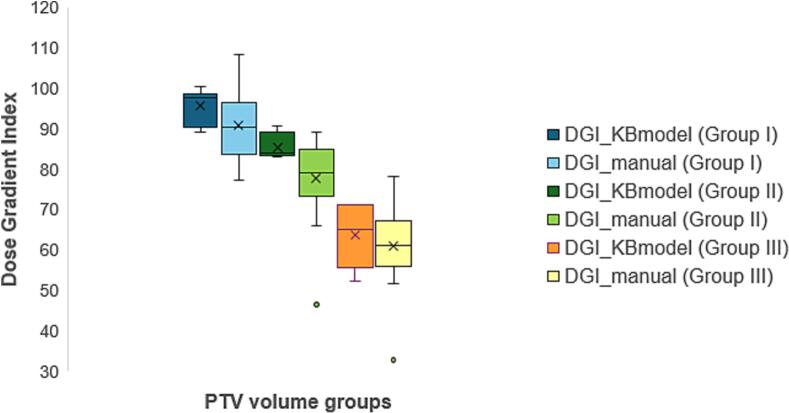
Comparison of DGI between KB optimized and manual plans for three different lesion groups.

The clinical implementation of the KB model reduced planning variability. A statistically significant reduction (p < 0.0001) was observed in CI, decreasing from 1.16 ± 0.19 in historical clinical plans to 1.08 ± 0.05. Similarly, when lesions were divided into three groups based on PTV volume (< 3 cm^3^, 3–10 cm^3^, and ≥ 10 cm^3^), improvements in DGI and inter-planner variability were also observed. Values of 95.7 ± 4.1 vs 90.7 ± 7.4, 85.4 ± 3.6 vs 77.7 ± 9, 63.6 ± 8.3 vs 60.9 ± 10.2 were found respectively for the clinical plans optimized based on the KB model against the manually optimized plans (Fig. 2).

By comparing the effective radius for different isodose values (85%, 65%, 50%, 40% and 30%), a significant reduction was obtained between the plan effective radius and the one predicted by the model (Table 4).

**Table 4:** Difference (with relative significance) between the radius predicted by the KB model and the effective radius obtained with plans.

R_eff_ Isodose	Model predicted (mm)	Clinical plan (mm)	Difference (mm)	p - value
85%	12.1 ± 5.3	11.8 ± 5.3	−0.3 (−1.3–0.2)	< 0.0001
65%	13.9 ± 5.8	13.5 ± 5.8	−0.4 (−1.1–0.1)	< 0.0001
50%	15.7 ± 6.4	15.1 ± 6.3	−0.6 (−1.1 - -0.1)	< 0.0001
40%	17.2 ± 6.9	16.5 ± 6.8	−0.7 (−1.6–0)	< 0.0001
30%	19.6 ± 7.8	18.9 ± 7.6	−0.8 (−1.9–0.3)	< 0.0001

A significant reduction of beams was found (102 ± 44 vs 146 ± 58; *p* = 0.002) for the KB model generated plans. Although not significant, a reduction was also found for the number of nodes (61 ± 23 vs 70 ± 27.4; *p* = 0.19) and treatment time (37 ± 11.9 min vs 41 ± 11.9 min; *p* = 0.17).

## 4 Discussion

In this study, a simple KB prediction model was developed and validated using a dataset of clinically approved intracranial radiosurgery treatment plans. The model demonstrated good predictive accuracy and robustness across the evaluated cases, resulting in treatment plans with clinically favorable dosimetric characteristics, with the most notable improvement observed in dose gradient metrics. The results suggest that clinically achievable plan quality can be effectively captured through a relatively simple predictive framework.

Similar to the method proposed by Yu et al. [22], the proposed approach associated the equivalent radius of the PTV with the equivalent radius of the volume receiving a specified percentage of the prescription dose. An excellent predictive accuracy (R^2^ > 0.97) was found for all considered isodose levels. Lower R^2^ values for lower isodose lines were likely associated with an expected larger plan variability. Based on the PTV volume, the linear fit can be used to guide planners in defining concentric isodose shells at predicted distances, thereby enabling improved control of dose fall-off.

This approach supported a more standardized and automated optimization process, improving planning efficiency while reducing the likelihood of suboptimal dose distributions. It is indeed true that the dose fall-off gradient is influenced by both the target size and the collimator size. In our study, the collimator sizes used in the KB plans were the same as those routinely employed in clinical practice, individually chosen by the planners to balance treatment time, dose gradient, and target conformity. This approach ensured that the gradient observed in the KB plans reflects clinical practice rather than an artificial model assumption. Therefore, although the collimator size contributes to the dose gradient, the correlation between dose fall-off and target size represents the combined, clinically relevant effect of target volume and collimator selection in a real-world scenario.

Both validations showed that the KB model produced automatic plans comparable or superior to expert manual plans. A higher DGI was found for KB-model plans, consistent with a steeper dose fall-off. A reduction of the irradiated healthy brain volume was found, particularly at low doses. The largest DGI improvement was specifically associated with sub-optimal clinical plans, highlighting the KB model's potential in quality standardization.

A comparable or superior dose conformality was also found together with higher efficiency, in terms of beams/nodes and delivery time reduction. Although not statistically significant, the greatest volume sparing was observed at the 30% isodose level.

Consistency with previous reports demonstrating the advantages of KB models in non-stereotactic [24], [25] and stereotactic settings [26], including improved plan quality, reduced planning time, and lower inter-planner variability, was observed. In stereotactic radiosurgery, Yu et al. [22] proposed a linear model to predict Gradient Measure (GM = r_50%_ - r_100%;_ where r_50%_ is the equivalent radius of the 50% prescription isodose volume and r_100%_ is the equivalent radius corresponding to the prescription isodose volume) and brain V_50%_ (brain volume that receives 50% of the prescription dose), achieving improved CI with minimal deviation from predicted values. Likewise, Hellerbach et al. [27] developed a model to estimate the lowest achievable V_12 Gy_ (brain volume receiving 12 Gy) in robotic radiosurgery.

Unlike these approaches, this shell-based model directly integrated dose gradient control into optimization, rather than using it only as a quality metric. A significant reduction in the time required to produce clinically acceptable plans was observed.

Even without additional manual refinement, fully automatic plans were generally clinically acceptable. Early clinical use showed the KB model complements, rather than replaces, clinical expertise, making optimization faster and more efficient.

Despite overall strong performance, the KB model exhibited limitations in anatomically atypical cases, particularly in the presence of multiple targets, targets adjacent to critical structures with complex geometries and very small lesions (< 0.3 cm^3^), where dose variability tends to be more pronounced. From the outset, the focus was on single lesions located at a safe distance from serial critical structures. For PTVs close to (serial) critical organs, optimization could not rely only on the model-predicted isodose shells, which assume a symmetric dose fall-off. Additional organ-specific dose constraints need to be applied, altering the dose distribution and making the linear relationship between target size and dose gradient not fully applicable. In such cases, the KB prediction serves only as baseline, and manual refinement is required for clinical acceptability.

Based on our early clinical experience, in the case of multiple targets, the KB model could be partially implemented by using the KB prediction for more isolated lesions and manually adding dose constraints for more irregular and complex targets. Considering that dedicated stereotactic treatment planning platforms do not offer automated planning techniques [28], [29], the proposed method could in principle be implemented everywhere, once adapted toward the locally available technology. Future developments could involve the extension toward a generalized KB model using data from multiple centers to broaden its applicability [15]. The same approach could also be extended to more complex cases, such as multiple brain lesions, and likely adapted to body SBRT applications.

In conclusion, the implementation of a KB model in radiosurgery proved to be feasible, advantageous, and safe, provided that rigorous validation criteria are met. With further validation the model could assist in clinical planning, capable of increasing efficiency and reducing variability, while still requiring careful and informed use by the clinical team.

## CRediT authorship contribution statement

**Rita Buono:** Data curation. **Roberta Castriconi:** Investigation, Data curation. **Alessia Tudda:** Data curation. **Carmen Gigliotti:** Investigation. **Lucia Perna:** Resources. **Paola Mangili:** Resources. **Antonella del Vecchio:** Supervision. **Claudio Fiorino:** Writing – review & editing, Writing – original draft, Supervision, Project administration. **Sara Broggi:** Writing – review & editing, Writing – original draft, Validation, Resources, Investigation, Formal analysis, Data curation.

## Declaration of competing interest

The authors declare that they have no known competing financial interests or personal relationships that could have appeared to influence the work reported in this paper.
