# Supplementary material for: Development and validation of a knowledge-based model for robotic radiosurgery planning for brain lesions
Source: Phys Imaging Radiat Oncol. 2026 Jun 11;39:101020. doi: 10.1016/j.phro.2026.101020 (PMC13315768; doi:10.1016/j.phro.2026.101020)
Supplement: Supplementary file 1 — Supplementary material [file mmc1.pdf]

Supplementary Material

Supplementary material A

In addition to Conformity Index (CI), the normalized conformity index (nCI) was calculated according to:

$$nCI = \frac{CI}{\%PTV100\%}$$

Where %PTV100% is the percentage of PTV volume encompassed by the 100% isodose. Compared to CI, nCI provides a more comprehensive evaluation of plan quality. While target coverage and dose gradient are typically assessed through separate metrics, the nCI incorporates information related to both adequate target coverage and the extent of dose spillage outside the PTV.

The results analyzed in terms of nCI were reported in the Tables.

	**nCi_Manual**	**nCi_KBmodel**	**p-value**
Internal Validation	1.11 ± 0.06	1.07 ± 0.03	0.004
External Validation	1.25 ± 0.17	1.11 ± 0.06	0.002

	**nCi**	**p-value**
Historical Plans (Manual)	1.19 ± 0.19	0.02
Clinical Plans (KB optimized)	1.08 ± 0.5	

Table S1: Geometric and dosimetric patients' data.

**Geometric parameters**	***Volume PTV (cm^3)***	6.99 cm^3 (0.18-43.85 cm^3)
	PTV-OAR minimum distance (mm)	40.66 mm (5.15-97.99 mm)
**Dosimetric parameters**	***Schedule (Dose/fractions)***	15 Gy/ 1fr 1 pts<br>18 Gy /1fr 1 pts<br>19 Gy/1fr 1 pts<br>24 Gy /1fr 31 pts<br>25 Gy/1fr 1 pts<br>25 Gy/5fr 3 pts<br>27 Gy/3fr 2 pts<br>27.5 Gy/5fr 1 pts<br>30Gy/3fr 11 pts<br>30 Gy/5fr 7 pts<br>35 Gy/5fr 1 pts
	Isodose Prescription	77.96 % (50 - 88.31 %)
	PTV_100% (%)	98.10 % (88.8- 100 %)
	Vbody_100% (cm^3)	7.66 cm^3 (0.2- 48.82 cm^3)
	Vbody_85% (cm^3)	10.94 cm^3 (0.35 - 66.87 cm^3)
	Vbody_65% (cm^3)	16 cm^3 (0.6 - 100.2 cm^3)
	Vbody_50% (cm^3)	22.53 cm^3 (0.92- 142.4 cm^3)
	Vbody_40% (cm^3)	29.71 cm^3 (1.27- 191.6 cm^3)
	Vbody_30% (cm^3)	43.6 cm^3 (1.94- 280.5 cm^3)

Table S2: Automatic optimization template.

Target Goals				
VOI	***Goal Type***	***Weight***	***Specified Dose (Gy)***	***Volume % / cm^3***
PTV	Minimum	50	Prescription dose	100 %
	Minimum	50	1 Gy less vs Prescription dose	99 %
	Maximum	10	120 % prescription dose	0
**Critical Goals**				
VOI	***Goal Type***	***Weight***	***Specified Dose (Gy)***	***Volume % / cm^3***
Shell 1 (Reff_85%)	Mean	1	85% prescription dose	0
Shell 2 (Reff_65%)	Mean	1	65% prescription dose	0
Shell 3 (Reff_50%)	Max	1	50% prescription dose	0
Shell 4 (Reff_40%)	Max	1	40% prescription dose	0
Shell 5 (Reff_30%)	Max	1	30% prescription dose	0
Shell 6 (40-50 mm)	Max	1	10% prescription dose	0

Table S3: Linear regression coefficients for different isodose curves: the slope coefficient (a), the intercept coefficient (b) and the coefficient of determination (R^2) are reported.

Isodose Level	a	b	R^2
**100%**	1.029	0.001	0.997
**85%**	1.112	0.063	0.994
**65%**	1.214	0.133	0.983
**50%**	1.328	0.193	0.984
**40%**	1.441	0.231	0.980
**30%**	1.621	0.284	0.977
